# Supplementary material for: Stability and asynchrony of local communities but less so diversity increase regional stability of Inner Mongolian grassland
Source: eLife. 2022 Oct 7;11:e74881. doi: 10.7554/eLife.74881 (PMC9545536; doi:10.7554/eLife.74881)
Supplement: Transparent reporting form [file elife-74881-transrepform1.docx]

***eLife’s* transparent reporting form**

We encourage authors to provide detailed information *within their submission* to facilitate the interpretation and replication of experiments. Authors can upload supporting documentation to indicate the use of appropriate reporting guidelines for health-related research (see [EQUATOR Network](http://www.equator-network.org/%20)), life science research (see the [BioSharing Information Resource](https://biosharing.org/)), or the [ARRIVE guidelines](http://www.plosbiology.org/article/info:doi/10.1371/journal.pbio.1000412) for reporting work involving animal research. Where applicable, authors should refer to any relevant reporting standards documents in this form.

If you have any questions, please consult our Journal Policies and/or contact us: [editorial@elifesciences.org](mailto:editorial@elifesciences.org).

**Sample-size estimation**

- You should state whether an appropriate sample size was computed when the study was being designed
- You should state the statistical method of sample size computation and any required assumptions
- If no explicit power analysis was used, you should describe how you decided what sample (replicate) size (number) to use

Please outline where this information can be found within the submission (e.g., sections or figure legends), or explain why this information doesn’t apply to your submission:

Sample size is clearly stated in the Materials and Methods section as well as in Figure 1.

**Replicates**

- You should report how often each experiment was performed
- You should include a definition of biological versus technical replication
- The data obtained should be provided and sufficient information should be provided to indicate the number of independent biological and/or technical replicates
- If you encountered any outliers, you should describe how these were handled
- Criteria for exclusion/inclusion of data should be clearly stated
- High-throughput sequence data should be uploaded before submission, with a private link for reviewers provided (these are available from both GEO and ArrayExpress)

Please outline where this information can be found within the submission (e.g., sections or figure legends), or explain why this information doesn’t apply to your submission:

Measurement frequency and replicates were clearly stated in the Materials and Methods section as well as relevant figures, such as Figure 1.

**Statistical reporting**

- Statistical analysis methods should be described and justified
- Raw data should be presented in figures whenever informative to do so (typically when N per group is less than 10)
- For each experiment, you should identify the statistical tests used, exact values of N, definitions of center, methods of multiple test correction, and dispersion and precision measures (e.g., mean, median, SD, SEM, confidence intervals; and, for the major substantive results, a measure of effect size (e.g., Pearson's r, Cohen's d)
- Report exact p-values wherever possible alongside the summary statistics and 95% confidence intervals. These should be reported for all key questions and not only when the p-value is less than 0.05.

Please outline where this information can be found within the submission (e.g., sections or figure legends), or explain why this information doesn’t apply to your submission:

Statistical analysis methods were clearly stated in the Materials and Methods section. Raw data and exact p-values were showed in figures, such as Figure 2 and 4. Besides, exact p-values can also be found in figure supplement files, such as those for Figure 2, 3 and 4.

(For large datasets, or papers with a very large number of statistical tests, you may upload a single table file with tests, Ns, etc., with reference to sections in the manuscript.)

**Group allocation**

- Indicate how samples were allocated into experimental groups (in the case of clinical studies, please specify allocation to treatment method); if randomization was used, please also state if restricted randomization was applied
- Indicate if masking was used during group allocation, data collection and/or data analysis

Please outline where this information can be found within the submission (e.g., sections or figure legends), or explain why this information doesn’t apply to your submission:

The Materials and Methods section clearly stated the method used to separate observed 21 filed sites (plant communities) into 10 different groups. Each group (referred to a regional community) contain 2 sites (with 1 remainder) and is independent with each other. This resampling procedure was repeated 1000 times resulting in 1000 resampled sets, each set containing 10 groups (regional communities). Then, these 1000 sets were analyzed with randomized examination. This is a restricted randomization as each resampled set has same sample size. This information can be found in the Materials and Methods section and Figure 1.

**Additional data files (“source data”)**

- We encourage you to upload relevant additional data files, such as numerical data that are represented as a graph in a figure, or as a summary table
- Where provided, these should be in the most useful format, and they can be uploaded as “Source data” files linked to a main figure or table
- Include model definition files including the full list of parameters used
- Include code used for data analysis (e.g., R, MatLab)
- Avoid stating that data files are “available upon request”

Please indicate the figures or tables for which source data files have been provided:

This study provided all relevant results in the appendix, figure supplement files and sources data files. In addition, we also uploaded the dataset, R codes and files summarizing details in analyses to Figshare (please see Data availability statement). Definitely, the dataset and R codes can also be uploaded as further supporting files if it is needed. Now, links to dataset and R code were shown in relevant figures, such as Figures 2–4 as well as their figure supplement files.
